# A Computational Model for Nme1Cas9 HNH Activation Driven by Dynamic Interface Engineering at Residues S593 and W596

**DOI:** 10.3390/biom16030358

**Published:** 2026-02-27

**Authors:** Zhenyu Zhou, Lizhe Zhu

**Affiliations:** Warshel Institute for Computational Biology, School of Medicine, The Chinese University of Hong Kong, Shenzhen 518172, China; zhenyuzhou@link.cuhk.edu.cn

**Keywords:** Nme1Cas9, molecular dynamics, HNH activation

## Abstract

Nme1Cas9 is an encouraging genome-editing tool with high fidelity and compactness, but its applications are limited by poor catalytic efficiency compared with SpyCas9. Understanding the dynamic activation mechanism of the HNH nuclease domain is the key to breaking the kinetic bottleneck. Here, we integrated Steered Molecular Dynamics (SMD) with the Traveling-Salesman-based automated Path Searching (TAPS) algorithm to reconstruct the atomic-level activation landscape of the L1-HNH module. The simulations suggest a complex “Lifting-Rearrangement-Sliding” pathway, revealing the critical role of a “Backbone Sliding” conformation; in this step, the HNH domain rotates across the R-loop surface. A thermodynamic analysis using free energy decomposition by MM/PBSA indicates that the intrinsic instability of the wild-type HNH/R-loop interface constitutes the predominant energetic barrier. Hyperactive variants (S593Q/W596K and S593Q/W596R) can overcome this barrier by substantially increasing binding affinity to the R-loop through a “Geometry–Electrostatics Synergism”: S593Q improves interfacial proximity, whereas W596K/R acts as an “Electrostatic Anchor.” The results of unbiased MD simulations demonstrate that strengthened interfacial interactions effectively promote spontaneous conformational drift toward the activated state. This computational study proposes a novel in silico model for “Dynamic Interface Engineering” in which reinforcing transient interfacial contacts during conformational sliding can be an effective strategy in developing high-efficiency CRISPR-Cas effectors.

## 1. Introduction

Clustered regularly interspaced short palindromic repeats (CRISPR) and CRISPR-associated (Cas) systems are advanced prokaryotic adaptive immune systems that are now repurposed as a powerful genome-engineering platform [1,2,3,4,5,6,7]. These systems were categorized into two broad classes based on their organization of the effector module: Class 1 systems, which depend on multi-subunit effector complexes, and Class 2 systems, which employ a single, multi-domain effector protein [8,9]. Due to their simplified architecture and programmable operation, Class 2 effectors have provided a new research template for applications ranging from basic biological studies to the clinical therapeutic correction of genetic disorders.

Within this increasingly large Class 2 toolbox, *Neisseria meningitidis* Cas9 (Nme1Cas9) emerges as a promising in vivo candidate. As a compact Type II-C effector consisting of only 1082 amino acids, Nme1Cas9 possesses a streamlined architecture that allows efficient “all-in-one” packaging into single adeno-associated virus (AAV) vectors, thereby circumventing a major delivery bottleneck in mammalian tissues [10,11,12,13,14]. In addition, Nme1Cas9 possesses extraordinary intrinsic fidelity, with minimal off-target cleavage, and can be finely regulated by natural anti-CRISPR proteins (Acrs), such as AcrIIC3, which serves as a potent off-switch by tethering Cas9 complexes from activating in a particular conformation [15,16,17,18]. With these essential therapeutic benefits in mind, Nme1Cas9’s general utility is currently limited by its kinetically unfavorable profile, namely its suboptimal intrinsic catalytic activity and cleavage efficiency compared to other widely used orthologs [16,19,20].

The induction mechanism of Nme1Cas9 is a complex process: upon complete R-loop (the tDNA-gRNA heteroduplex) formation, the RuvC and REC2 domains and the L1 linker are subject to large-scale conformational transitions, which induce the HNH domain to escape from its inactive interface with RuvC, make a substantial rotation towards the RNA-DNA heteroduplex, and finally dock near the cleavage site on the target strand [20,21,22]. Recent comprehensive reviews and computational studies emphasize that such precise conformational checkpoints and allosteric communications are universal master regulators directing the nuclease activity across diverse CRISPR-Cas systems [23,24]. Given the large scale of this conformational excursion of the L1-HNH domain and its underlying structural instability, it is impossible to observe this dynamic process directly using a static structural technique; thus, detailed mechanistic information about the domain rearrangement remains elusive. More importantly, in such a complex enzymatic system, it is commonly observed that the rate-limiting step is governed by the large-scale conformational changes rather than the chemical reaction itself [25]. Thus, a straightforward elucidation of these dynamic processes is a prerequisite for achieving systematic regulation of Cas cleavage efficiency. Indeed, recent advanced molecular dynamics simulations have successfully captured the dynamic interactions and conformational barriers restricting the final activation of the HNH nuclease domain in other Cas9 orthologs, highlighting the power of integrated computational approaches [26]. In particular, pioneering computational studies by Giulia Palermo and co-workers have profoundly reshaped our understanding of Cas9 dynamics. Their extensive molecular dynamics simulations have revealed the striking structural plasticity of the HNH domain and elucidated the long-range allosteric communication networks that govern its activation in SpyCas9 Palermo’s work [27,28] demonstrated that HNH activation is not merely an isolated rigid-body swing, but rather a highly correlated process intricately coupled with the motions of the REC lobe and the non-target DNA strand [29]. These milestone studies underscore the necessity of treating Cas9 as a dynamic ensemble and inspire the application of enhanced sampling techniques to capture elusive conformational intermediates.

Earlier experiments by Sun et al. [20] have suggested that stabilizing the HNH domain in its activated state by strengthening HNH-R-loop interactions is a reasonable strategy to enhance the activation efficiency of Nme1Cas9. Following this logic, mutants bearing S593Q/W596K and S593Q/W596R mutations were previously created and rigorously validated through in vitro DNA cleavage assays by Sun et al., resulting in significantly improved catalytic performance that rivals SpyCas9. Here, however, we show that the functional role of residues S593 and W596 extends beyond stabilizing the final active conformation. Using Steered Molecular Dynamics (ABMD from plumed and enforced rotation from Gromacs) [30,31,32] Molecular Dynamics (MD)-based Traveling Salesman-Based Automated Path Searching (TAPS) [33,34,35,36], we reconstructed the dynamic trajectory of the L1-HNH module after complete R-loop pairing. We characterized the sequence of rising, rearrangement, and sliding motions leading to the activated conformation. In the initial lifting process, the HNH domain exits from its inhibitory interface with the RuvC domain. Following L1-HNH reconfiguration, the HNH domain re-approaches the R-loop and, steered by electrostatic guidance residues, rotationally slides along the R-loop surface to finally dock into the catalytic conformation. Importantly, we find that residues S593 and W596 interact with the phosphate backbone of the R-loop as early as this rotation-sliding step. Confirming this observation through extensive unbiased MD experiments and MM-PBSA [37] free energy calculations, we show that these mutations substantially increase the binding affinity to the R-loop during this dynamical transition, facilitating the sliding mechanism of the L1-HNH module and thermodynamically increasing the likelihood of the enzyme to navigate the energy landscape to the active state.

In this paper, we present an all-atomistic simulation of the L1-HNH activation process after complete R-loop pairing and clarify the exact mechanistic functions of residues S593 and W596 and their variants S593Q/W596K and S593Q/W596R in the process of the key rotational sliding conformational transition, by identifying that a stronger binding affinity at the L1-HNH/R-loop interface during sliding favors the transition of the global structure into the activated state, we find a direct causal relation between the stabilization of the intermediate state and the enhancement of the activation efficiency. Our work also demonstrates that the rational design of complex nucleases controlled by large-scale conformational dynamics need not be restricted to static structural templates, but can be efficiently guided by mechanistic understanding derived from dynamical molecular paths.

## 2. Materials and Methods

### 2.1. System Construction and Force Field Parameters

The initial models for Nme1Cas9 were constructed based on high-resolution crystal structures. The seed-paired ternary complex (PDB ID: 6KC7) served as the initial state, and the catalytically poised complex (PDB ID: 6JDV) was employed as the final state. Missing residues and disordered loops were modeled using MODELLER (Version 10.5, University of California San Francisco, San Francisco, CA 94143, USA) [38] to ensure structural continuity. Mutant systems (S593Q/W596R and S593Q/W596K) were generated via the PyMOL (Version 3.1.6.1, Schrödinger, LLC, New York, NY, USA) [39] mutagenesis wizard.

Each protein-nucleic acid complex was solvated in a cubic box of TIP3P [40] water molecules with a 10 Å buffer. The systems were neutralized and further ionized with KCl and MgCl_2_ to achieve final concentrations of 100 mM KCl and 10 mM MgCl_2_.

### 2.2. Molecular Dynamics (MD) Simulations

All MD simulations were performed using GROMACS 2019.4 (University of Groningen, Groningen, The Netherlands) with the Amber14SB-OL15 [41,42] force field to describe molecular interactions. Energy minimization was conducted 10,000 steps of steepest descent followed by the conjugate gradient algorithm to remove steric clashes. After minimization, the system was equilibrated in two stages: NVT ensemble at 300 K for 1 ns to equilibrate the solvent and NPT ensemble for 1 ns at 1 atm, controlled using the Berendsen barostat algorithm.

MD simulations used a 1 fs time step with periodic boundary conditions (PBC). Long-range electrostatic interactions were treated using the Particle Mesh Ewald (PME) method, while short-range electrostatics and van der Waals interactions were handled with a 10 Å cutoff. The LINCS algorithm was applied to constrain all bonds.

### 2.3. Initial Path Generation

To ensure structural stability and accuracy, the initial transition path was generated using a reverse pulling strategy. The starting and ending conformations for this procedure were derived from 50 ns conventional MD simulations initiated from the catalytically poised state (PDB ID: 6JDV) and the seed-paired complex (PDB ID: 6KC7), respectively. Given that the HNH domain and L1 linker are fully resolved in the 6JDV structure while other regions in 6KC7 are partially incomplete, the activation trajectory was sampled by driving the system from the 6JDV activated state back toward the 6KC7 configuration.

The enhanced sampling combined Enforced Rotation in GROMACS and ABMD in PLUMED (version 2.5.3, SISSA, Trieste, Italy). The Enforced Rotation module was utilized to drive the HNH-L1 domain away from the catalytic site with an isotropic rotation rate of 0.045 deg/ps and a force constant of 500.0 kJ/(mol·nm^2^) over a 2 ns simulation. The rotation vector was set to (−0.608, −1.858, −0.883) with a pivot point at (3.3494, 6.8489, 4.2994). To further refine the trajectory, a 2ns ABMD simulation was implemented with a high force constant (KAPPA) of 100,000 kJ/(mol·nm^2^). The RMSD of all heavy atoms in the L1 linker and HNH domain served as the collective variable, with alignment performed on the Ca atoms of helical regions in the RuvC, REC1, REC2, and WED domains.

The preliminary trajectory for subsequent path optimization was assembled by concatenating the 2 ns ABMD and 2 ns enforced rotation segments. This integrated path effectively captures the conformational transition between the two functional states and serves as the baseline input for the TAPS path optimization protocol.

### 2.4. Path Optimization

The TAPS (Traveling-Salesman-Based Automated Path Searching) method was utilized to refine the initial transition path into the low free energy path (LFEP). The theoretical background and methodological details of TAPS have been previously documented in our published work. Path optimization was performed using a custom Python (version 3.5, Python Software Foundation, Wilmington, DE, USA) script (https://github.com/liusong299/TAPS, accessed on 12 December 2025). The convergence of the optimized pathway was validated through PCV-$\sqrt{\left\langle z\right\rangle }$ analysis, as illustrated in Figure S6.

### 2.5. Binding Free Energy Calculation

Binding free energies between the HNH domain and the fully paired R-loop (comprising the gRNA and target DNA) were calculated along the optimized MFEP using the MM-PBSA (Molecular Mechanics Poisson-Boltzmann Surface Area) protocol. The calculations were performed for every frame (interval = 1) using the MMPBSA.py module in AmberTools [43]. The Amber14SB force field and OL15 parameters were employed for the protein and nucleic acids, respectively. To account for the highly charged nature of the DNA-containing system, the GB-Neck2 (igb = 8) implicit solvent model was utilized with a salt concentration of 0.15 M. Crucially, the internal dielectric constant (intdiel) was set to 10, a value recommended for protein-nucleic acid complexes to better capture the screening effects of the polarizable environment.

## 3. Results

### 3.1. Dynamic Landscape of L1-HNH Activation

**Figure 1:**
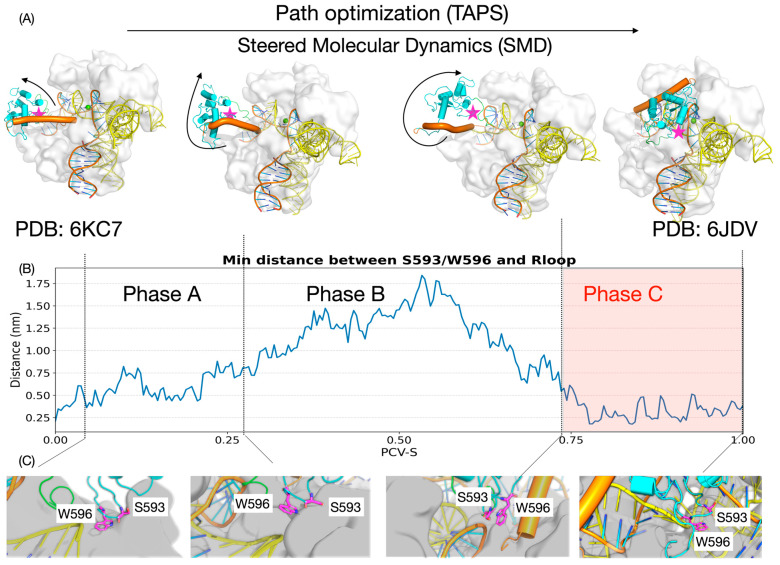
Dynamic activation landscape of the Nme1Cas9 HNH domain revealed by TAPS path optimization. (**A**) Key structural snapshots along the optimized activation pathway. The reaction path was initially generated using Steered Molecular Dynamics (SMD)—specifically combining PLUMED’s ABMD and GROMACS’s enforced rotation module—and subsequently refined via TAPS path optimization on path collective S (PCV-S) [44]. The sequence illustrates the transition from the inactive state (based on PDB ID: 6KC7) to the final activated state (based on PDB ID: 6JDV), involving domain lifting, L1-HNH rearrangement, and rotation along the R-loop surface. The HNH domain is colored in cyan, the L1 linker in orange, and the sgRNA in yellow. The target DNA strand is depicted with an orange backbone and blue bases. The spatial locations of critical residues S593 and W596 are marked with magenta stars. (**B**) Minimum distance between the mutation sites (S593/W596) and the R-loop backbone along the optimized trajectory. (**C**) Detailed structural close-ups of the local environment around S593 and W596 corresponding to the states shown in figure. Residues S593 and W596 are rendered as magenta sticks, and the L2 loop is highlighted in green. The color scheme for the L1 linker (orange), HNH domain (cyan), and nucleic acids (yellow/orange/blue) remains consistent with (**A**).

We approximated the full-fledged atomic-level dynamic path of Nme1Cas9 going from its inactive R-loop-pairedits its state to active state using the path optimization algorithm TAPS. To ensure that the optimized pathway was not biased by the relatively large force constant used in the initial Steered MD (SMD) guess, we evaluated the convergence of the trajectories across the iterative TAPS optimization process. As depicted in the Multidimensional Scaling (MDS) projection (Figure S7), the initial aggressive pulling pathway (iter000) systematically relaxed and migrated across the conformational landscape. Ultimately, the pathways from the final iterations (e.g., iter130–137) converged into highly identical trajectories. This convergence quantitatively confirms that the non-equilibrium artifacts from the initial SMD were completely eliminated, yielding a thermodynamically robust, intrinsic low free-energy path (LFEP) for subsequent mechanistic analyses. Activation of HNH domain is a highly non-trivial process. By analyzing the changes in the smallest distance between the important mutation sites (S593/W596) and the R-loop backbone in combination with the spatial displacement and rotation properties of the HNH domain alone the PCV-S [44], we segmented the HNH allosteric activation process, which is a complex L1-HNH allosteric activation, into three kinetic phases (Phase A–C) (Figure 1A).

#### 3.1.1. Phase A: Domain Lifting and Steric Release

In the initial phase of activation, the HNH domain undergoes a “lifting” motion. During this process, the HNH gradually lifts and slips from its initial contact interface with RuvC domains in the inactive state. This directional displacement is of significant importance, as it effectively eliminates the steric hindrance around the HNH domain and produces the conformational room needed for subsequent large-scale rotational movement of L1-HNH. In the meantime, the distance between S593/W596 and the R-loop gradually expands from 2 Å, which means that the weak interaction between them is very unstable, making L1-HNH in a state of high freedom (Figure 1A–C).

#### 3.1.2. Phase B: Conformational Rearrangement and L1 Restructuring

As the process proceeds, the L1-HNH module proceeds into the next rearrangement phase. We found that the L1-HNH domain begins to exhibit small-scale rotational changes in the process of finding the correct binding orientation. In this process, the minimum distance between S593/W596 and the R-loop exhibits a non-monotonic “expansion-contraction” behavior. It is worth highlighting that such process is accompanied by significant changes in the secondary structure of L1 linker—the L1 takes a special angle of bending and α-Helix folding/bending changes. Such a rigidification of the L1 alpha-helix structure not only limits the random vibration of HNH but also functions as a coiled spring that directs the HNH domain to approach and rotate accurately toward the target R-loop (Figure 1A–C).

#### 3.1.3. Phase C: Electrostatic Sliding and Subsequent Docking (The Decisive “Backbone Sliding” Stage)

During the final, most decisive stage of activation, the HNH domain reaches the completion of large-scale rotation. In contrast to the “lifting” and detachment of Phase A, this stage involves the re-establishment of intimate interfacial contacts between the L1-HNH module and the R-loop. In this phase, the distance between S593/W596 and the R-loop is reduced and stabilized, oscillating within a narrow range of 2–5 Å. This suggests that the L1-HNH domain does not merely “jump” to the active site but instead follows a sophisticated “sliding-rotation” mechanism. Under the electrostatic guidance of surface residues (including S593 and W596), the domain slides along the heteroduplex backbone and gradually optimizes its orientation to overcome the final energetic barrier. This intimate “Backbone Sliding” process is pivotal for locking the HNH domain into the catalytic conformation and accurately orienting its active site toward the scissile phosphodiester bond. As this final phase determines the specificity and cleavage efficiency of the enzyme, the dynamic processes and energy characteristics within this Phase C represent the primary focus of the further mutational and thermodynamic studies.

### 3.2. Energetic Profile and Critical Metastable Intermediate Analysis During Activation of the HNH Domain

**Figure 2:**
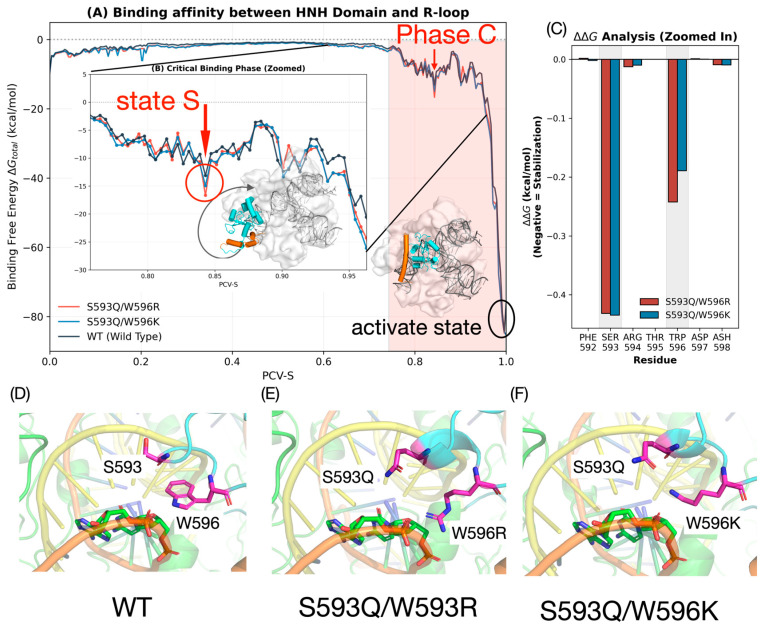
Thermodynamic characterization of the HNH activation pathway and stabilization of the intermediate State S. (**A**) Binding free energy (∆G_total_) profiles between the HNH domain and the R-loop along the TAPS-optimized activation trajectory PCV-S. The Wild-type (WT) is shown in black, the S593Q/W596K variant in blue, and the S593Q/W596R variant in red. The structural inset displays the final Activated State, with the HNH domain colored in cyan and the L1 linker in orange. (**B**) A zoomed-in view of the critical binding phase (nested within (**A**)), highlighting the local energy minimum identified as “State S.” The structural inset illustrates the conformation of the L1-HNH module at this intermediate state (HNH in cyan, L1 in orange). (**C**) Per-residue binding free energy difference (∆∆G) analysis for the mutation sites and adjacent residues. Values are calculated as ∆∆G = ΔG_mut_ − ΔG_WT_ where negative values indicate enhanced stabilization relative to the WT. Blue bars represent S593Q/W596K, and red bars represent S593Q/W596R. (**D**–**F**) Detailed structural comparison of the binding interface at State S for (**D**) WT, (**E**) S593Q/W596R, and (**F**) S593Q/W596K. Residues 593 and 596 are rendered as magenta sticks. The R-loop complex is depicted with an orange backbone and green bases for DNA, and yellow for sgRNA. Note the closer proximity and favorable orientation of the mutant residues toward the DNA backbone compared to the WT.

To further explain the thermodynamic driving force for improving the activation efficiency of HNH domain mediated by S593Q and W596R/K mutations, we estimated the time-dependent binding free energy (∆G_total_) between HNH domain and the R-loop complex along the TAPS-optimized dynamic route by the MM/PBSA method. From the energy profile (Figure 2A), a key dynamic property of this process can be seen, in the track of the HNH domain from the inactive state to the active state (activation progress). In phase A, the binding energy between L1-HNH and the R-loop gradually rises near 0. In this process, the contact between HNH and the R-loop constantly decreases. In the subsequent phase B, when L1-assisted rotation of L1-HNH occurs, because HNH is very flexible at this stage, it is far from contact with the R-loop and surrounding protein domains, and the binding energy still stays near 0. In phase C, when HNH recontacts the R-loop and starts the large-angle sliding process of L1-HNH on the R-loop surface, the binding energy decreases as contact between L1-HNH and the R-loop increases, to roughly −80 kcal/mol. In phase C, there is a local minimum in the binding energy. We have defined that as the key metastable intermediate “State S”. In the critical State S stage, the binding energy advantage of the mutants is significantly enhanced, and the binding energy of S593Q/W596R and S593Q/W596K is roughly 2 kcal/mol lower than WT (Figure 2B). The residue energy decomposition of the whole activation process (∆∆G = ΔG_mut_ − ΔG_WT_) also showed that this energy reduction can specifically be ascribed to the contribution of positions 593 and 596 to the binding energy after mutation (Figure 2C, Figures S1 and S2). To understand the structural basis of this energy discrepancy, we extracted representative conformations of State S for interface analysis. In State S, the loop region of the HNH domain (sites 593/596) is spatially very close to the DNA phosphate backbone of the R-loop, which is a critical window in establishing intermolecular interactions. However, in the WT system, the side chain of S593 is too short and, because of the geometric constraints, it cannot reach the DNA backbone to a form good interaction with it; at the same time, although W596 had huge side chain volume, the electrostatic attraction between its indole ring’s N-H groups with the negatively charged phosphate backbone is very weak and unstable: hence, it is a very poor binding in the WT system in this state (Figure 2D).

After the introduction of the mutation, the interaction pattern at this interface changed qualitatively. The S593Q mutation appended a longer side chain to glutamine (Gln), effectively bridging the interface and pulling the residue much closer to the DNA backbone, thereby forming electrostatic or hydrogen-bond networks. In the case of site 596, the substitutions of W596R/K added a strong positive charge. Compared to the neutral tryptophan, the positively charged lysine (Lys) and arginine (Arg) residues produced extreme electrostatic attractions with the negatively charged DNA backbone, like an “electrostatic anchor” for the HNH domain anchoring R-loop. In particular, the W596R mutation, with the complex guanidino backbone from multiple angles, thereby significantly reducing the energy barrier to the conformational transition and stabilizing the active-state conformation (Figure 2E,F).

To rigorously verify that this identified State S is a genuine metastable intermediate rather than a transient artifact of the enhanced sampling process, we subsequently subjected these structures to long-timescale unbiased MD simulations (500 ns × 2), as detailed in the following Section 3.3.

### 3.3. Spontaneous Conformational Variant Drift Towards Activated State

**Figure 3:**
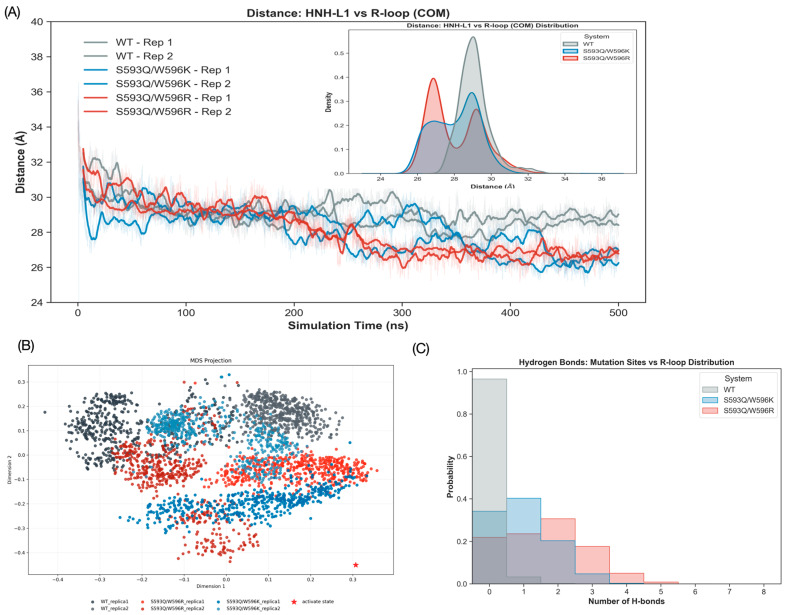
Spontaneous evolution toward the activated state in unbiased MD simulations. (**A**) Time evolution (**left**) and probability distribution (**right**) of the L1-HNH Center-of-Mass (COM) distance between State S and final activated State. Mutant variants show a distinct sub-population at a shorter distance (~2.7 nm). (**B**) 2D MDS projection of the HNH conformational ensemble. The red star indicates the reference activated state. (**C**) Number of hydrogen bonds between residues 593/596 and the R-loop backbone. S593Q/W596R (red) shows the highest occupancy.

To confirm the dynamic characteristics of the variant state “State S” over a long period of simulation time and to explore whether the mutation provides the complex with an intrinsic driving force for the evolutionary transition towards the final activated state, we deduced representative conformations of State S along the TAPS pathway. We conducted unbiased molecular dynamics simulations (500 ns × 2 replicas) for both WT and the two mutant systems. Although State S is still far from the fully activated state in terms of space conformation (requiring considerable rotation of L1-HNH) compared with the fully activated state defined by the crystal structure, the RMSD analysis of the HNH domain against the activated state (Figure S5) demonstrated dramatic differences. We selected all heavy atoms of L1-HNH to calculate the RMSD. Results showed that the RMSD of the WT system remained high throughout the simulation; that is, the system was typically maintained in this intermediate state, whereas, with no external bias in the forcing terms, the RMSD of both S593Q/W596R and S593Q/W596K systems exhibited a spontaneous, slow-decreasing trend. This conformational drift trend was intuitively confirmed in the multidimensional scaling (MDS) projection of the L1-HNH heavy atoms. The conformational ensemble of the mutants was significantly closer to the reference point representing the activated state (red asterisk) than that of WT (Figure 3B).

To quantify the spatial proximity effect, we examined the dynamic trend of the change in the center-of-mass (COM) distance between the center of the L1-HNH domain and the center of the R-loop. As the simulation time increased, the center distance of the mutant systems showed a dynamic decrease, gradually moving towards the compact activated state conformation, reaching 2.6 nm at the end of the trajectory, whereas that of WT fluctuated around 3.0 nm, demonstrating no obvious directional migration (Figure 3A). Furthermore, the probability density distribution of the center-of-mass distance (Figure 3A) helps quantify this difference. In addition, the WT shows a single peak (~3.0 nm) in its distribution. In contrast, the two mutants show a bimodal distribution, with a substantial subpopulation of formations at a shorter distance (~2.7 nm). These suggest that the contribution via interaction initiated by the mutation effectively reduces the conformational energy barrier, enabling the HNH domain to explore and lock into a spatial position closer to the activated state.

The above dynamic tendency and its underlying molecular mechanism are further illustrated in the hydrogen bond analysis and secondary structure stability of the L1 linker (Figure 3C, Figures S3 and S4). The number of hydrogen bonds between the 593/596 position and the R-loop is almost zero in the WT system; thus, the indole ring N-H group of 596 has difficulty maintaining a stable contact with the DNA backbone in the dynamic environment. In contrast, the mutant systems form a stable hydrogen-bonded topology. Particularly, the S593Q/W596R system constructs many more hydrogen bonds than the S593Q/W596K system. When merged with the above-mentioned MMPBSA energy analysis, this result further confirms that the guanidinium head of the arginine (Arg) side chain is of superior geometrical arrangement and has a multi-directional capacity of electrostatic interaction with lysine (Lys), in a stronger manner, can easily grasp the DNA backbone when sliding around HNH, driving conformational adjustments. Structurally, this transition is facilitated by the rigidification of the L1 linker (Figure S3). While WT trajectories exhibit largely disordered coil structures reflecting high intrinsic flexibility, the mutants maintain a continuous ∂-helical conformation. This implies that the variants transform the L1 linker into a stable mechanical element to facilitate HNH domain activation.

## 4. Discussion

### Nme1Cas9 as a High-Fidelity Editor: Mechanistic Insights and Rational Design

The unique properties of Nme1Cas9—especially its small size, high fidelity, and regulatable activity—make this Cas9 variant a promising next-generation gene-editing tool. However, its broader application has been limited by relatively low catalytic efficiency compared with the mature SpyCas9 system [10,11,12,13,14,15,16,17,18,19,20,21,22]. Bridging this gap requires a fundamental understanding of the dynamic activation mechanisms that regulate HNH domain transitions. Here, we used Steered Molecular Dynamics (SMD) in conjunction with the TAPS path optimization algorithm to divulge for the first time the complete atomic-level conformational landscape governing the L1-HNH module’s transition between the R-loop-bound inactive state and the catalytically competent state.

The simulation results depict a sophisticated “Lifting-Rearrangement-Sliding” activation pathway, in which the HNH domain first gains steric freedom via domain lifting, then undergoes L1-HNH structural rearrangement, and finally undergoes a critical “Backbone sliding” motion along the R-loop surface. To fully contextualize these findings, it is instructive to compare this activation landscape with the well-characterized mechanism of *Streptococcus pyogenes* Cas9 (SpyCas9). In SpyCas9, it is well established that the HNH domain undergoes a massive allosteric rearrangement. As elegantly mapped by Palermo’s group through network analysis and MD simulations, the SpyCas9 HNH domain relies on a global, long-range allosteric communication network spanning the REC lobe and the R-loop to reach its catalytically competent state [29,45]. Therefore, the fundamental requirement for large-scale HNH mobility is a conserved feature across Cas9 orthologs. However, the specific “Lifting-Rearrangement-Sliding” trajectory characterized in our study appears to be uniquely tuned to the structural idiosyncrasies of Nme1Cas9. Due to its highly compact architecture and distinct linker compositions compared to SpyCas9, Nme1Cas9 exhibits a more restricted conformational space. Consequently, it relies heavily on specific, transient electrostatic interactions—such as the “Geometry–Electrostatics Synergism” mediated by residues S593 and W596 during the “Backbone Sliding” phase—to precisely navigate its unique conformational energy landscape.

By integrating MM/PBSA free-energy decomposition, we quantified the energetic evolution of the HNH-R-loop interface during the process and identified the crucial roles of residues S593 and W596. We demonstrate that the intrinsic weakness of the interaction between these wild-type residues and the DNA backbone constitutes a substantial energetic barrier to activation. Moreover, we present a robust mechanistic explanation for the enhanced cleavage efficiency of the S593Q/W596K and S593Q/W596R variants, showing how these mutations provide a “Geometry–Electrostatics Synergism”. While S593Q finely tunes interfacial distance, W596K/R acts as an “Electrostatic Anchor.” The MD simulation results also indicated that these strengthened interactions do not merely stabilize the complex but serve as a kinetic driver, driving a spontaneous conformational drift of the L1-HNH domain towards the activated state. Interestingly, we found that the Arginine variant (W596R) functioned as a more effective “Molecular Gear,” leveraging its bidentate hydrogen-bonding ability to promote smooth sliding. At the same time, the mutation-provided rigidity of the L1 linker afforded the required mechanical support for this transition.

This study is not only valuable for providing theoretical insights into the activation mechanisms of CRISPR-Cas systems but also establishes a new computational model for the rational design of high-efficiency Nme1Cas9 variants. According to these findings, future engineering efforts should no longer focus solely on static affinity but also on pursuing a strategy of “Dynamic Interface Engineering”: specifically, strengthening the binding interactions between L1-HNH and the R-loop during the sliding process. By reinforcing this dynamic interface, the entropic penalty of conformational search can be effectively reduced, guiding the HNH domain more smoothly into its catalytic registry. As with any in silico mechanistic model, subsequent studies will extend this design approach to other potential sites and validate the proposed mechanisms through detailed in vitro cleavage assays and in vivo editing experiments.

It should be noted that the absolute binding free energy values calculated via MM/PBSA in this study do not include conformational entropy contributions and may therefore appear exaggerated. However, because our primary focus is on the relative energetic differences (ΔΔG) between structurally similar WT and mutant variants, the entropic contributions are assumed to be largely comparable, making the relative enthalpy-driven trends robust and informative.

## 5. Conclusions

Based on our in silico simulations, we reconstructed the atomic-level “Lifting-Rearrange-Sliding” activation pathway of Nme1Cas9 using TAPS path optimization, revealing that an essential backbone sliding motion of L1-HNH along the R-loop is mandatory for catalytic competence. Thermodynamic analysis showed that the S593Q/W596K and S594Q/W596R variants strengthen this process via “Geometry–Electrostatics Synergism,” with strengthened interfacial contacts promoting the HNH domain towards its active conformations.

We explicitly emphasize that the proposed activation pathways and energetic mechanisms constitute a simulation-based interpretive model. While rigorously supported by thermodynamic calculations, these computational hypotheses warrant further experimental validation via advanced structural and in vivo techniques.

## Data Availability

The original contributions presented in this study are included in the article/Supplementary Materials. Further inquiries can be directed to the corresponding author.

## Supplementary Materials

The following supporting information can be downloaded at https://www.mdpi.com/article/10.3390/biom16030358/s1, Figure S1: Energy component decomposition reveals distinct stabilization mechanisms for mutant residues; Figure S2: Ranking of top contributing residues to interfacial binding energetics in Wild-type and variant HNH domains.; Figure S3: Time-evolution of secondary structure stability in the L1 linker; Figure S4: Evolution of interfacial hydrogen bond networks during MD simulations; Figure S5: RMSD evolution of the HNH domain relative to the activated crystal structure (500 ns × 2); Figure S6: Convergence test by calculation for optimized path; Figure S7: Convergence validation of the L1-HNH activation pathway during TAPS optimization; Table S1: Details of ABMD; Table S2: Details of TAPS.

## Abbreviations

The following abbreviations are used in this manuscript:

NmeCas9	*Neisseria meningitidis* Cas9
TAPS	A traveling-salesman based automated path searching method
L1	Linker 1 helix
HNH	Linear dichroism
