# Supplementary material for: A Computational Model for Nme1Cas9 HNH Activation Driven by Dynamic Interface Engineering at Residues S593 and W596
Source: Biomolecules. 2026 Feb 27;16(3):358. doi: 10.3390/biom16030358 (PMC13024101; doi:10.3390/biom16030358)
Supplement: Supplementary file 1 [file biomolecules-16-00358-s001.zip › biomolecules-4117325-supplementary.pdf]

Supplementary Materials

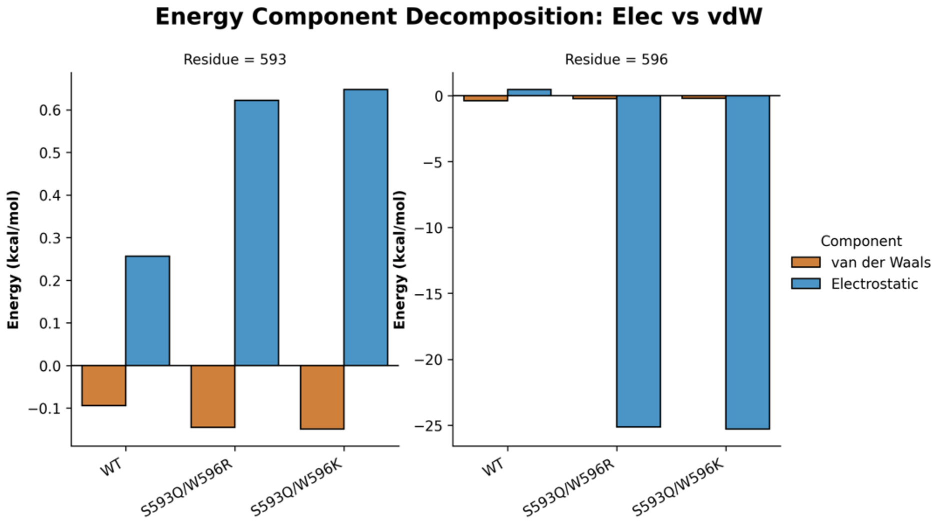

Figure S1. **Energy component decomposition reveals distinct stabilization mechanisms for mutant residues.** Decomposition of the per-residue binding free energy into Electrostatic (ΔE_elec , blue bars) and van der Waals (ΔE_vdw, orange bars) contributions for the critical mutation sites 593 and 596. (Left Panel, Residue 593) Comparison between WT (Ser) and mutants (Gln). The S593Q mutation displays enhanced interaction energies, primarily attributed to optimized van der Waals contacts and polar interactions, supporting the "Geometric Fine-tuning" hypothesis where the longer Glutamine side chain bridges the interfacial gap. (Right Panel, Residue 596) Comparison between WT (Trp) and mutants (Arg/Lys). The substitution of the hydrophobic Tryptophan with positively charged Arginine or Lysine results in a substantial favorable contribution from the electrostatic term (prominent blue bars), confirming the "Electrostatic Anchor" mechanism where the cationic side chains establish strong salt-bridge-like interactions with the anionic R-loop backbone. Note that S593Q/W596R (Arg) exhibits a comparable electrostatic profile to S593Q/W596K (Lys), consistent with its robust binding affinity.

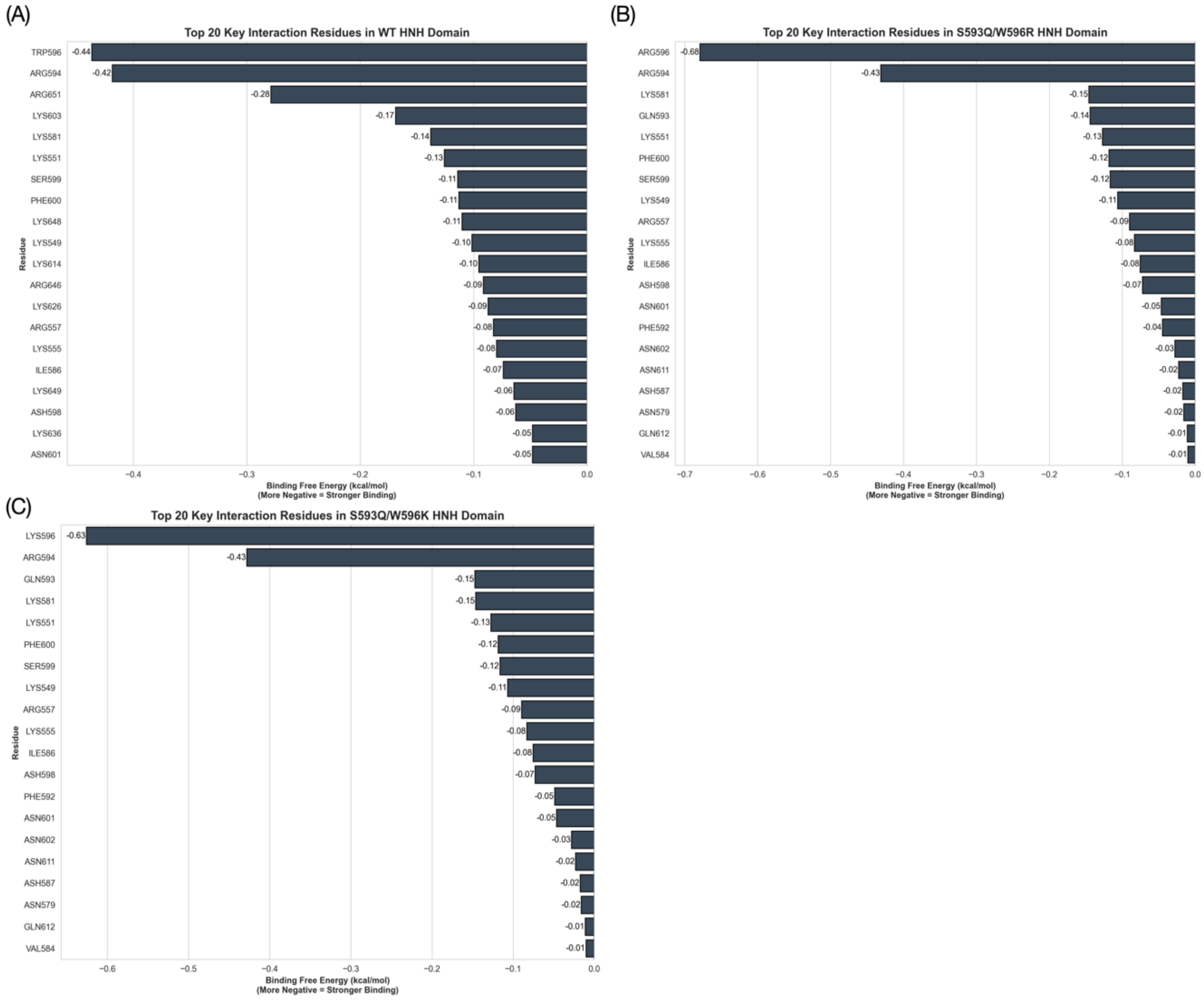

Figure S2. Ranking of top contributing residues to interfacial binding energetics in Wild-type and variant HNH domains. Based on MM/PBSA per-residue free energy decomposition, the top 20 amino acids making the most favorable energetic contributions to R-loop binding are displayed for (**A**) WT, (**B**) S593Q/W596R, and (**C**) S593Q/W596K systems. Horizontal bars represent the total binding free energy (kcal/mol) for each identified residue. More negative values indicate stronger interactions and greater contributions to complex stabilization.

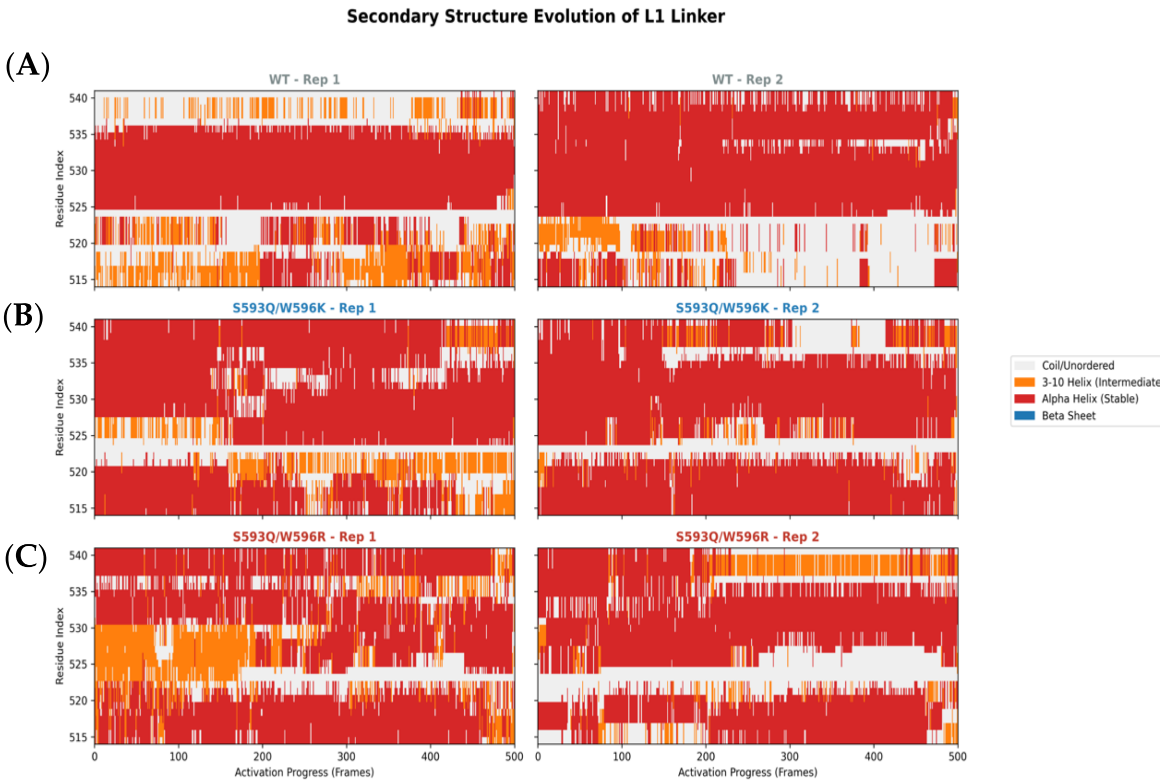

Figure S3. Time-evolution of secondary structure stability in the L1 linker. Secondary structure content of the L1 linker region was monitored over 500 ns MD simulations using the DSSP algorithm. Color coding: α-helix (red), 3/10-helix(orange), coil (white), and ß-sheet (blue). (**A**) Wild-type (WT): Trajectories exhibit largely disordered structures dominated by coil regions (white) with only fragmented and transient helical segments (red), reflecting the high intrinsic flexibility of the native L1 linker. (**B**–**C**) Variants (S593Q/W596R and S593Q/W596K): In contrast, the mutant trajectories display a relative stabilization of secondary structure, maintaining a continuous ∂-helical conformation (red) throughout the simulation, with minor transitions to 3/10 helix (orange) or Coil (white). This mutation-induced rigidification suggests that the variants transform the L1 linker into a stable mechanical element to facilitate HNH domain activation.

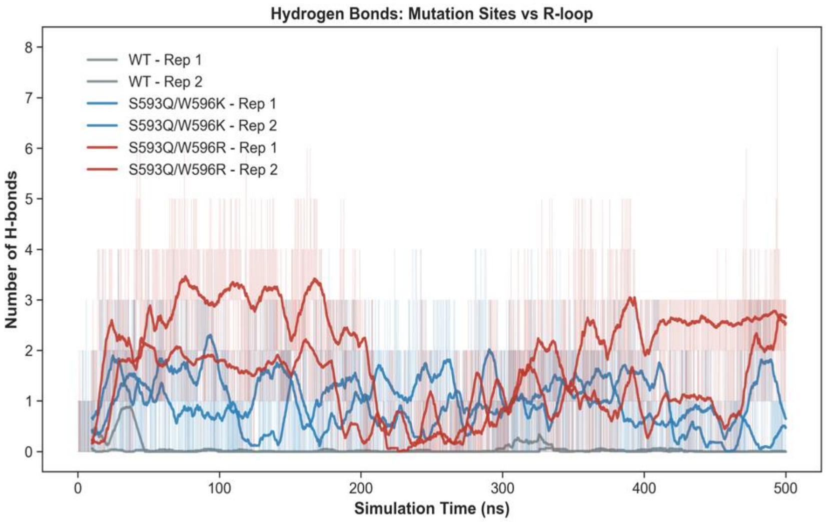

Figure S4. Evolution of interfacial hydrogen bond networks during MD simulations. The number of hydrogen bonds between the critical residues (593/596) and the R-loop phosphate backbone is plotted against simulation time (500ns). Color Coding: Wild-type (WT) is shown in gray, S593Q/W596K in blue, and S593Q/W596R in red. Darker and lighter shades correspond to two independent simulation replicas. Data Visualization: Semi-transparent background lines depict the raw data frequency, while bold opaque lines represent the smoothed running average. Observation: The WT system exhibits negligible hydrogen bonding (near 0)In contrast, the S593Q/W596R variant establishes the most robust interaction network (averaging ~2–3 bonds), supporting the hypothesis that the Arginine side chain facilitates multi-point contacts with the DNA backbone more effectively than Lysine.

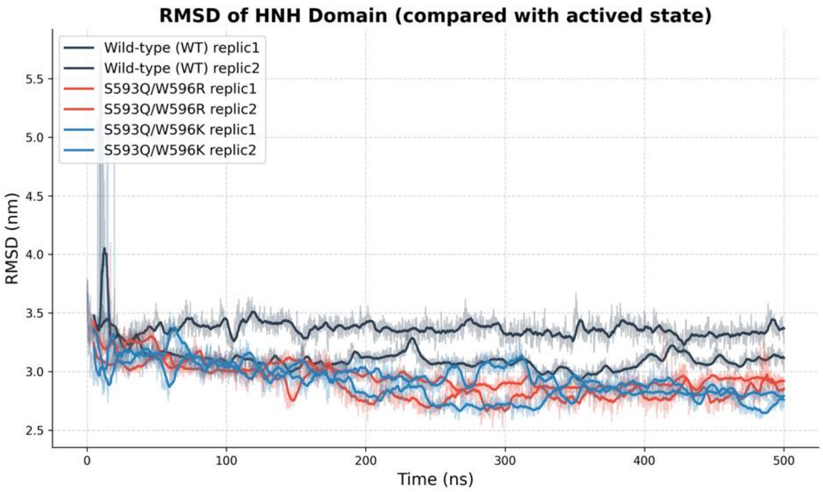

Figure S5. **RMSD evolution of the HNH domain relative to the activated crystal structure (500 ns×2).** Color coding: WT (black/grey), S593Q/W596K (blue/light blue), and S593Q/W596R (red/dark red). Lighter and darker lines represent two independent replicates. Notably, the variant trajectories (red and blue) exhibit a spontaneous decrease in RMSD values over time, indicating a structural convergence toward the active conformation compared to the WT.

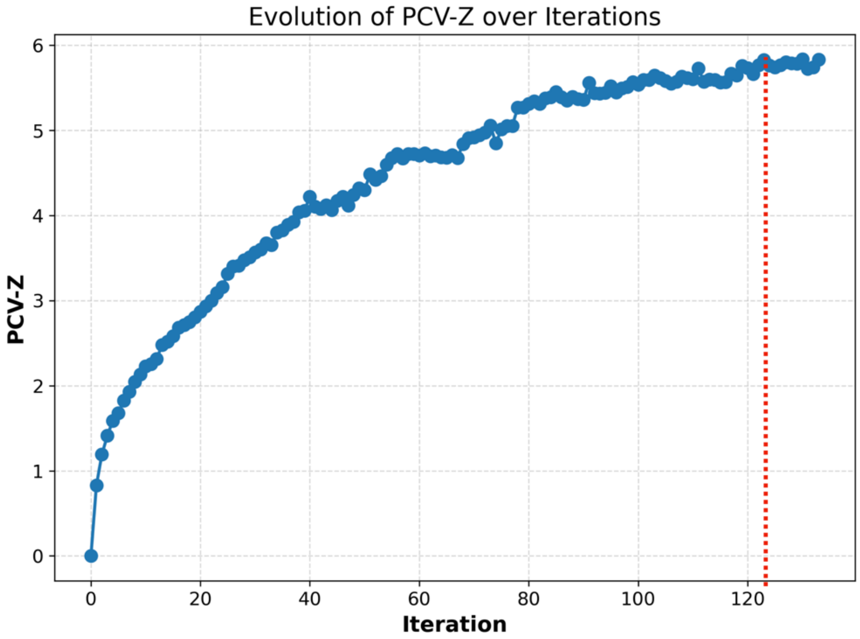

Figure S6. **Convergence test by calculation for optimized path.** Convergence of the optimization processes for the paths is measured by the progress of along the simulation iteration. The initial paths are the reference. Convergence iteration is highlighted by a red dashed line.

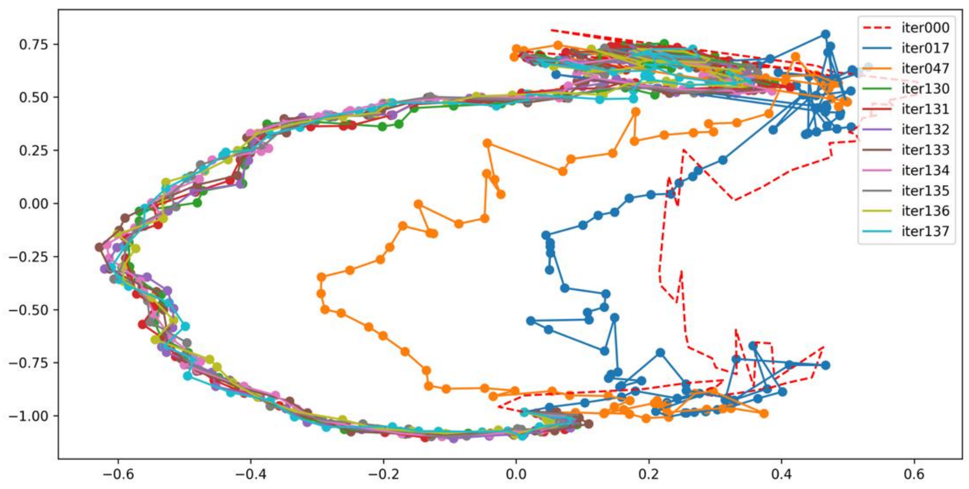

Figure S7. Convergence validation of the L1-HNH activation pathway during TAPS optimization. The 2D Multidimensional Scaling (MDS) projection illustrates the conformational evolution of the transition pathway across different optimization iterations. The initial guess pathway (iter000) generated by Steered Molecular Dynamics (SMD) with a large force constant. The highly overlapping trajectories in the final iterations (e.g., iter130 to iter137) demonstrate that the pathway has converged to a low free-energy path systematically relaxed through the TAPS algorithm, effectively eliminating any non-equilibrium artifacts or biases introduced by the initial strong pulling force.

Table S1. Details of ABMD

Initial Path	WT-Nme1Cas9 activate
Initial State	PDB ID: 6JDV (active state of NmeCas9), equilibrated after 50 ns MD simulation
Target State	PDB ID: 6KC7 (seed-paired ternary complex)
Temperature	300k
Pressure	1 atm
Force constant	100,000 kJ/(mol·nm²)
Frame Record Frequence	10 ps
Total Sampling Time	2 ns
Atom sets	RMSD: all heavy atoms in the L1 linker and HNH domain
	Alignment: Ca atoms of helical regions in the RuvC, REC1, REC2, and WED domains

Table S2. Details of TAPS.

Optimized Path		WT-Nme1Cas9 activation
Sampling Time of Each Iteration		4000 ps
Temperature		300 K
Atoms Set	Alignment	Cα of helical part in RuvC, REC1, REC2 and WED domain
	RMSD	Heavy atoms of L1 Linker and HNH [illegible]
Tolerant Distance for Neighbor Nodes		1.4 Å
Well-tempered Metadynamics simulation	Gaussian Height	0.25 kJ/mol
	Gaussian Width	0.5
	Bias Factor	10
Length of tMD		10 ps
Force Constant of tMD		150,000 kJ/mol/nm2
Frame Record Frequence		1 ps
